# Screening of agronomic and qualitative physical and chemical traits of 83 naked oats strains

**DOI:** 10.1371/journal.pone.0324879

**Published:** 2025-05-27

**Authors:** Longlong Liu, Zihao Wei, Jia Li, Jincheng Peng, Shengsheng Tang, Yanzhen Wang, Mingchuan Ma, Zhang Liu, Shirui Xu

**Affiliations:** 1 Center for Agricultural Genetic Resources Research, Shanxi Agricultural University, Taiyuan Shanxi, China; 2 College of Agriculture, Shanxi Agricultural University, Jinzhong Shanxi, China; 3 Germplasm Enhancement on Loess Plateau, Ministry of Agriculture and Rural Affairs, Taiyuan Shanxi, China; 4 Hou Ji Laboratory in Shanxi Province, Taiyuan Shanxi, China; Institute of Genetics and Developmental Biology Chinese Academy of Sciences, CHINA

## Abstract

Naked oats (*Avena nuda*) are a key cereal crop that are valued for their high nutritional content and desirable agronomic traits. In this study, 80 developed strains and three bred varieties were evaluated in Kelan County, Shanxi Province, China, from 2022 to 2023. Six agronomic, eight physical, and five chemical traits were assessed. Significant phenotypic variations were observed across the 83 strains, with marked improvements in agronomic traits in 2023, particularly in grain shape, thousand grain weight, and spike length. The grain shape showed the most substantial improvement, whereas the thousand grain weight remained relatively stable. Cluster analysis identified three agronomic groups, with Group III comprising high-yield, stable strains, such as YM49. Regarding the physical traits, a general reduction in grain size and fullness was observed in 2023, with hardness exhibiting the greatest variation. Principal component analysis classified the strains into three morphological groups, with YM23 representing a large-grain, soft-textured type. Chemical quality traits indicated an increase in β-glucan and water content in 2023, whereas fat content decreased while exhibiting high variability. Nutritional profiling clustered the strains into three groups, with YM39 leading in protein, β-glucan, and fat content. Correlation analysis across the selected 19 traits highlighted the key tradeoffs between grain morphology, yield, and nutritional quality. Effective tillering was identified as a crucial trait that influences the overall performance. Strain YM49 demonstrated a consistent high-ranking performance across multiple traits, indicating its potential for breeding high-quality oats with high yields.

## Introduction

Naked oats (*Avena nuda*) are a widely cultivated crop, valued as both a forage grass and a food source, especially in temperate regions [1,2]. They are highly nutritious, low in sugar, rich in essential minerals and vitamins, and provide high biomass yield and excellent forage for livestock [3]. In recent years, with increasing awareness of the nutritional and feed value of naked oats, the demand for breeding new and improved varieties is growing [4].

Despite the economic significance of naked oats, breeding efforts are hampered by limited germplasm evaluation. This study aimes to bridge this gap by assessing key traits associated with yield and quality [5]. The primary objective of improving naked oat is to develop high-yielding and high-quality varieties. Germplasm resources serve as the genetic foundation for crop improvement by delineating a diverse pool of candidate genes for breeding [6]. Elucidation of the genetic diversity of naked oat germplasm is crucial for understanding the available genetic resources and leading the selection of elite varieties [7]. The systematic collection, identification, and evaluation of naked oat germplasm facilitate the selection of varieties with desirable agronomic and quality traits, while also enhancing our understanding of the genetic diversity within this species [8]. Therefore, it is essential to assess the key traits and adaptability of naked oat germplasm to support breeding.

Yield and quality traits are critical parameters in naked oat breeding. The key biological factors influencing yield include spike length, tillering number, and thousand grain weight, all of which contribute to plant growth, vigor, and overall yield potential [9]. Genetic improvement of these traits remains a major goal in oat breeding programs [10,11]. Understanding the diversity of agronomic traits provides valuable insights into the adaptability, productivity, and breeding potential of naked oat strains [12]. A comprehensive evaluation of existing varieties is essential for targeted genetic improvement and selection of superior breeding materials.

Grain quality has become a key focus in naked oat breeding, particularly for improving both physical and chemical attributes. These traits influence processing performance and nutritional value, making them critical targets for food and feed applications. Among the chemical components, protein, fat, β-glucan, and starch contents are especially important due to their relevance to human health and industrial utility [3,13,14].

Naked oat varieties have been extensively studied their morphology [15], cytology [16], physiology and biochemistry [17], and molecular characteristics [18,19]. Morphological identification is the most direct and cost-effective method yet for germplasm screening and trait selection. However, the application of molecular techniques in oat breeding is increasing. Naked oat germplasm exhibits high genetic diversity and significant trait variation, providing a strong basis for breeding of new and improved oat varieties [20,21].

In this study, we evaluated 80 crossbred strains and three inbred varieties of naked oats cultivated in Kelan County, Shanxi Province, China, during the 2022–2023 growing season. Agronomic and quality traits were analyzed using correlation analysis, principal component, membership function analysis, cluster and grey correlation analyses. These analytical methods allowed the comprehensive evaluation of all 83 naked oat strains. Our results provide a solid basis for identifying germplasm with desirable agronomic and nutritional characteristics, thereby supporting breeding efforts aimed at improving both the yield and quality of naked oats cultivars.

## Materials and methods

### Experimental site

The test site for determining the traits of oat varieties is located in an oat-planting region of Xihui Village, Kelan County, Xinzhou City, Shanxi Province, China (111.61°*E*, 38.76°*N*; elevation 1,405 *m*). This area is a semi-arid, rainfed agricultural region with a mid-temperate, continental monsoon climate. The average annual temperature is 6°C, the average annual frost-free period is 120 days, the average annual precipitation is 456 *mm*, and the average annual sunshine duration is 2,752 hours (meteorological data sourced from the local Meteorological Bureau). Chernozem soils are present at the oat-planting site (soil data from the local Planning and Natural Resources Bureau). Throughout the oat-growing period (June-September), the mean monthly temperature in 2022 and 2023 was consistent with those of previous years. However, the mean monthly precipitation in July 2022 was significantly higher than in July 2023 or in previous years (S1 Table). The experimental site was located on agricultural land managed by Shanxi Agricultural University and designated for long-term research purposes, and no specific permits were required.

### Test materials

We tested 83 strains of naked oats obtained from the Center for Agricultural Genetic Resources Research at the Shanxi Agricultural University, Taiyuan City, Shanxi Province, China. Except for the varieties BY-13, BY-3, and JY-8, all other strains (80 varieties) were stable strains developed by Shanxi Agricultural University (Table 1).

**Table 1:** Names of naked oat varieties included in our trial.

Code	Name of strain	Code	Name of strain	Code	Name of strain
1	YM2	29	YM32	57	YM63
2	YM3	30	YM33	58	YM65
3	YM5	31	YM34	59	YM67
4	YM6	32	YM35	60	YM68
5	YM7	33	YM36	61	BY-13
6	YM8	34	YM37	62	YM71
7	YM9	35	YM38	63	YM72
8	YM11	36	YM39	64	YM73
9	YM12	37	YM41	65	YM74
10	YM13	38	YM42	66	YM75
11	YM14	39	YM43	67	JY-8
12	YM15	40	YM44	68	YM76
13	YM16	41	YM45	69	YM77
14	YM17	42	YM46	70	YM78
15	YM18	43	YM47	71	YM79
16	YM19	44	YM48	72	YM80
17	YM20	45	YM49	73	YM81
18	YM21	46	YM50	74	BY-3
19	YM22	47	YM51	75	YM82
20	YM23	48	YM52	76	YM83
21	YM24	49	YM53	77	YM84
22	YM25	50	YM55	78	YM85
23	YM26	51	YM56	79	YM87
24	YM27	52	YM57	80	YM91
25	YM28	53	YM58	81	YM92
26	YM29	54	YM59	82	YM94
27	YM30	55	YM61	83	YM96
28	YM31	56	YM62		

### Experimental design

All strains in the trial were sown in randomized blocks on May 18, 2022, and again on May 21, 2023. Each strain was replicated three times, with each replication consisting of an 8-row plot that was 7 m long, with 25 cm row spacing, resulting in a plot area of $14\;{\text{m}}^{2}$ (0.25 m × 8 × 7). Phosphorus (${\text{P}}_{2}{\text{O}}_{5}$) $525\;{\text{kg/hm}}^{2}$, urea (${\text{CH}}_{4}{\text{N}}_{2}\text{O}$) $150\;{\text{kg/hm}}^{2}$, and potassium sulfate (${\text{K}}_{2}{\text{SO}}_{4}$) $300\;{\text{kg/hm}}^{2}$ were added to the soil during its preparation. Artificial trench sowing was performed at a depth of 4-6 cm. A density of approximately 4.5 million seedlings per hectare was maintained. Weeding was performed at tillering and jointing stages, and other management measures followed local practices. After ripening, harvesting, and threshing, the seeds were washed, placed in labeled kraft paper bags, and dried at 37°C for 24 hours in an oven.

### Measuring agronomic traits

#### Thousand grain weight.

To measure thousand grain weight (TGW) in grams, two 1,000 seed samples were collected from each of three plots for each strain and weighed using a balance (Denver SI-234, Denver Instrument, USA) [22].

#### Productive tillers/plant.

Productive tillers/plant (PTP) was visually determined for 10 randomly selected plants in each of the three plots for each strain. The average number of tillers per plant was calculated as the PTP of each strain per plot.

#### Spike length.

The lengths of 10 randomly selected main spikes from each of three plots for each strain were measured using a straight ruler.The spike length (SL) was measured from the first whorl at the base of the spike to the top of the spikelets (excluding awns). Average length (*cm*) was recorded as the SL of the strain per plot.

#### Primary spikelets/spike.

The number of spikelets on each of the 10 main spikes was counted visually, and the mean value was recorded as the primary spikelets/spike (PPS) for the strain per plot.

#### Grains/spike.

Ten main spikes were randomly selected from each of the three plots for each strain and the grain number was determined for each of the ten main spikes from each plot after threshing. The average number of grains per spike was recorded as the grains/spike (GS) for each plot for a given strain.

#### Grain yield per unit area.

All harvested grains from each plot were threshed and allowed to dry naturally until the water content was less than 13%. The total grain harvested from each plot per strain was then weighed, and the average weight was used to calculate the yield per unit area (*kg*/*ha*; YA) [23].

### Qualitative physical traits of grain

#### Grain dimensions.

The physical dimensions of the grain, measured in millimeters (mm) or square millimeters (${\text{mm}}^{2}$) for two-dimensional measurements, were recorded for five traits: (1) length (GL), (2) width (GW), (3) area (GA), (4) perimeter (GP), and (5) length/width ratio (L/W). For each strain, these dimensions were measured in 300 mature seeds with 100 seeds collected from each plot. The seeds were randomly selected after harvest from the bulk sample of the entire yield from the plot of a given variety using an automatic seed testing analyzer (TPKZ-1, Zhejiang Topu Yunnong Technology Co., Ltd., Hangzhou, Zhejiang, China). This process was repeated thrice. Measurements were performed in three batches (300 seeds each), evaluating 900 seeds per strain.

#### Test weight of seeds.

To determine the test weight (kg, TW) of seeds, seed samples of 2000 *g* were collected from each of three plots for each strain after harvest. Seeds were randomly selected from the bulk sample of the combined yield from the three plots. The initial 2000-*g* sample of seeds was then divided into two 1000-*g* samples. Each 1000-*g* seed sample was screened four times using two sieves with diameters of 4.5 mm and 1.5 mm to remove both large and small debris, respectively. Seeds were screened in batches of approximately 250 *g*, and the debris were discarded. The screened seeds were then poured into the upper, middle, and lower chambers of a weight-measuring device [24]. The weight of each 1000-seed subsample was measured for both halves of the original sample. This process was repeated three times, resulting in six 1000-seed subsamples.

#### Grain specific gravity.

Fifteen gram seed samples from each strain were collected from each of the three plots. The mass (*W*_1_, *g*) of a clean, dry 50 mL bottle was measured using an electronic balance with 1% accuracy. Thereafter, 15 g of seeds from each strain were placed in the bottle and weighed (*W*_2_, *g*). Distilled water was added to the bottle until the seeds were completely covered. The bottle was gently shaken to remove any bubbles from the surface of the seeds, and water was added until it levelled with the 50-mL mark. The total mass of the seed-water solution was recorded (*W*_3_, *g*) [25]. The specific gravity of the grain (SG, ${\text{g\textbackslash{},cm}}^{-3}$) was calculated using the formula: SG = $W_{2}/[50-(W_{3}-W_{2}-W_{1}$)]. This process was repeated three times.

#### Seed hardness.

A sample of 300 seeds for each strain, consisting of 100 seeds from each plots, was selected from the bulk sample. The seeds were placed in a grain characteristics tester (SKCS 4100, Perten, Sweden; Shenzhen Qiushan Trading Co., Ltd., Shenzhen, China), and the average value of the 300 tests was used to determine the degree of hardness (HD).

### Qualitative chemical traits of grain

A 50-*g* sample of seeds from the original plot of each strain was placed in the detection tank of a near-infrared grain analyzer (Infratec TM 1241, FOSS; Shanghai Ruifen International Trade Co., Ltd., Shanghai, China). The fat content (% FC), protein content (% PC), total starch (% TSC), and water (% WC) contents were determined using an available oat model [26]. This process was repeated thrice. These replicates were obtained from the pooled grains of the three plots per treatment from which the samples were selected.

β-Glucan content (% β-gC) was measured from the same three samples using an enzyme assay [27], followed by a near-infrared assay [28]. The enzymatic assay was conducted using the standard glucan determination method (AACC Method 32-23), published by the American Cereal Chemists’ Society (AACC), following the commercial kit instructions from Megazyme (Shanghai Jinpan Biotechnology Co., Ltd., Shanghai, China). Seeds of each strain were calibrated using a near-infrared analyzer (Tensor37 Fourier Transform Infrared Spectrometer; INVENIO, Bruker Optics, Germany; Brooke Technology Co., Ltd., Beijing, China). β-gC levels in each sample were estimated using the predictive model for this trait.

### Statistical analysis

For the 83 strains tested each year, data for the six agronomic traits (TGW, PTP, SL, PSS, GS and YA), eight physical traits (GL, GW, GA, GP, L/W, TW, SG, and HD), and five chemical traits (FC, PC, TSC, WC, and β-gC) were calculated to obtain mean value ($\overset{―}{X}$), maximum value (Max), minimum value (Min), standard deviation (σ), and coefficient of variation (CV).

To assess differences among the 83 strains, data for each of the 19 measured traits were first subjected to square root transformation to improve normality. Thereafter, the Shapiro–Wilk test was applied to evaluate the distribution of transformed data for each trait. For traits that met the assumption of normality, a one-way analysis of variance (ANOVA) was performed to determine statistically significant differences among strains. For traits that violated the normality assumption, the non-parametric Kruskal–Wallis H test was used as a robust alternative to ANOVA.

The 19 traits was classified into ten stages based on the average value ($\overset{―}{X}$) and standard deviation (σ), with each stage representing a 0.5-σ interval. This method, which is widely used in genetic evaluations for breeding, facilitates the clear differentiation of trait variation. The 0.5-σ interval has been commonly applied in oat research and has proven effective in distinguishing phenotypic groups. While alternative approaches, such as quartiles or percentiles, could offer additional insights, the 0.5-σ threshold was selected for its established utility in breeding-related trait classification [29–31]. The stages were calculated as follows, based on the $\overset{―}{X}$ and σ values: [*X*_1_ < ($\overset{―}{X}$- 2.0σ)], [($\overset{―}{X}$-2.0σ) < *X*_2_ < ($\overset{―}{X}$-1.5σ)], [($\overset{―}{X}$-1.5σ) < *X*_3_ < ($\overset{―}{X}$-1.0σ)], [($\overset{―}{X}$-1.0σ) < *X*_4_ < ($\overset{―}{X}$ -0.5σ)], [($\overset{―}{X}$-0.5σ) < *X*_5_ < ($\overset{―}{X}$)], [($\overset{―}{X}$) < *X*_6_ < ($\overset{―}{X}$+0.5σ)], [($\overset{―}{X}$+0.5σ) < *X*_7_ < ($\overset{―}{X}$+1.0σ)], [($\overset{―}{X}$+1.0σ) < *X*_8_ < ($\overset{―}{X}$+1.5σ)], [($\overset{―}{X}$+1.5σ) < *X*_9_ < ($\overset{―}{X}$+2.0σ)] and [($\overset{―}{X}$+2.0σ) < *X*_10_]. The relative frequency (*P*_*i*_) of each stage was used to calculate the genetic diversity index ($H^{\prime }$), with $H^{\prime }=-\sum P_{i}\times \ln P_{i}$, where *P*_*i*_ represents the proportion of materials in the ${\text{i}}^{\text{th}}$ stage relative to the total number of materials [32].

Correlation and grey relational analyses were performed for all traits in each strain for each year. For the average values of all traits, correlation and principal component analysis (PCA), membership function, hierarchical clustering, and grey relational analyses were applied across both years.

To reduce multicollinearity in the PCA calculations, trait pairs with absolute Pearson correlation coefficient greater than 0.8, calculated based on the average values across both years, were examined for redundancy. For each highly correlated pair, one trait was excluded to retain a set of relatively independent variables. This preliminary step improved the robustness of PCA and enhanced the interpretability of the resulting components.

The fuzzy membership function was used to calculate the membership value for each trait [33] by positioning each trait within a closed interval [0, 1]. The function is expressed as: $U(X_{i})=(X_{i}\;-\;X_{min})/(X_{max}\;-\;X_{i})$, where *X*_*i*_ is the membership value of the ${\text{i}}^{\text{th}}$ trait, and *X*_*min*_ and *X*_*max*_ are the minimum and maximum trait values across all strains, respectively. The genetic distance between varieties was calculated using the squared Euclidean distance in the clustering analysis.

All 19 traits across the 83 strains were evaluated using grey system theory, with a simulated “ideal variety” constructed based on defined breeding goals and production requirements. To determine the optimal trait values, a grey relational analysis (GRA) was employed in combination with multi-objective optimization techniques, including the entropy weight method. This integrative approach enabled the identification of genotypes that best approximated the ideal variety by comprehensively balancing multiple traits and accounting for inherent tradeoffs, such as the compromise between grain yield and protein content. The primary objective was to optimize the overall performance across traits rather than maximize any single attribute in isolation [34,35]. Each trait was regarded as a factor in the system. The degree of correlation among all the factors in the system was analyzed. The greater the degree of correlation, the higher the comprehensive evaluation index and the degree of similarity among factors, indicating a high degree of similarity between a given strain or trait and an ideal strain or trait [36]. The series composed of the best values was considered the reference series *X*_0_, and the series composed of each trait index was considered the comparison series *X*_*i*_ (where *X* represents different strains and *i* represents different trait indicators) and *I* = 1, 2, 3,... .n, and *X*_0_ = { *X*_0_ (1), *X*_0_ (2), *X*_0_ (3), ... .*X*_0_ (n)}, *X*_*i*_ = { *X*_*i*_ (1), *X*_*i*_ (2), *X*_*i*_ (3), ... . *X*_*i*_ (n)}. All traits were sorted using a weighted coefficient, and the degree of correlation between the 83 strains and the “ideal strain” was determined to obtain the order (from good to bad) of each strain. The calculation formulae were as follows:

(1) Correlation coefficient:

$$\epsilon_{i}(k)=\frac{min_{i}min_{k}|X_{0}(k)-X_{i}(k)|+\rho max_{i}max_{k}|X_{0}(k)-X_{i}(k)|}{\left|X_{0}(k)-X_{i}(k)|+\rho max_{i}max_{k}|X_{0}(k)-X_{i}(k)\right|}$$ (1)

where $\epsilon_{i}(k)$ is the correlation coefficient of *X*_0_ and *X*_*i*_ at point *k*. $\Delta_{i}(k)=|X_{0}(k)-X_{i}(k)|$ represents the difference between the reference and comparison series at point *k*. ρ represents the resolution coefficient and ranges from 0 to 1. $min_{i}min_{k}|X_{0}(k)-X_{i}(k)|$ and $max_{i}max_{k}|X_{0}(k)-X_{i}(k)|$ indicate the minimum and maximum values between each trait and its corresponding reference series, respectively.

(2) Equal weight correlation degree:

$$R_{i}=\frac{\sum \epsilon_{i}(k)}{N}$$ (2)

where *R*_*i*_ represents the equal-weight correlation degree, and *N* represents the number of observations in the series.

(3) Weight coefficient:

$$\omega_{I}=\frac{R_{i}}{\sum R_{i}}$$ (3)

(4) Weighted correlation degree:

$$R_{i}^{\prime }=\frac{\sum \epsilon_{i}(k)\times \omega_{I}}{N}$$ (4)

All data were analyzed with SPSS Statistics 19.0 for Windows (IBM, Armonk, NY, USA).

## Results

### Agronomic traits

#### Variability and genetic diversity.

Significant differences were observed between the 83 strains for each trait (Table 2). The average values of TGW, SL, PSS and GS were higher in 2023 than in 2022, with GS showing the largest increase and TGW exhibited the smallest. In 2022, the CV for six traits ranged from 10.83% to 99.41%, with the following order of variation from highest to lowest: PTP > GS > PSS > YA > TGW > SL. The highest CV for PTP was observed in strain YM47 (2). SL had the lowest CV, with YM57 exhibiting the highest SL value (21 *cm*) and YM71 the lowest (11.2 *cm*). The genetic diversity indices for these six traits ranged from 1.8765 to 2.085, in the following order from highest to lowest: YA > GS > TGW > PSS > SL > PTP. In 2023, the CV for these six traits ranged from 11.1% to 149.8%, with the order of variation from highest to lowest being PTP > GS > PSS > YA > SL > TGW. The highest CV was observed for PTP, with YM81 having the highest value (1.8). TGW had the lowest CV, with strain YM56 exhibiting the highest value (40.13 *g*) and BY-13 the lowest (19.43 *g*). The genetic diversity indices for these six traits ranged from 1.3614 to 2.0147, and the order of traits from the highest to lowest was: PSS > GS > TGW > YA > SL > PTP.

**Table 2:** Variation in and genetic diversity of agronomic and quality traits in 83 naked oat strains during 2022 and 2023.

Traits		Year	Average	Min	Max	SD	Coefficient of variation (%)	Genetic diversity	Statistics		
									*F/H*	df	*P*
Agronomy	Thousand grain weight (*g*)	2022	24.52	16.89	35.34	3.13	12.77	1.999	240.617	82	< 0.05
		2023	29.63	19.43	40.13	3.3	11.13	2.0089	233.707	82	< 0.05
	Productive tillers/plant	2022	0.45	0	2	0.44	99.41	1.8765	136.281	82	< 0.05
		2023	0.32	0	1.8	0.47	149.76	1.3614	154.258	82	< 0.05
	Spike length (*cm*)	2022	15.5	11.2	21	1.68	10.83	1.9472	128.851	82	< 0.05
		2023	14.33	11	20.9	1.94	13.53	1.9711	157.508	82	< 0.05
	Primary Spikelets/Spike	2022	28.4	14.6	46.2	6.44	22.69	1.9592	148.275	82	< 0.05
		2023	28.89	13.2	52.8	7.79	26.96	2.0147	140.663	82	< 0.05
	Grains/Spike	2022	63.28	29	123.4	17.01	26.87	2.0036	3.567	248,82	< 0.05
		2023	62.83	17.2	120.2	18.43	29.33	2.0134	4.229	248,82	< 0.05
	Yield per area (*kg*/*ha*)	2022	3.13	1.49	4.99	0.63	20.11	2.085	22.72	248,82	< 0.05
		2023	3.03	1.28	4.22	0.6	19.79	1.9972	191.208	82	< 0.05
Physical quality	Grain length (*mm*)	2022	7.08	5.79	7.85	0.44	6.24	1.9844	233.898	82	< 0.05
		2023	7.42	5.96	8.27	0.53	7.11	1.9921	235.958	82	< 0.05
	Grain width (*mm*)	2022	2.1	1.84	2.51	0.13	6.12	2.0149	235.043	82	< 0.05
		2023	2.31	1.91	2.63	0.13	5.48	2.0213	220.932	82	< 0.05
	Grain area (${\text{mm}}^{2}$)	2022	11.27	8.86	14.08	1.06	9.41	2.0718	15.247	248,82	< 0.05
		2023	12.96	8.66	15.38	1.19	9.16	2.0352	215.79	82	< 0.05
	Grain perimeter (*mm*)	2022	16.47	13.81	17.83	0.89	5.43	1.943	232.982	82	< 0.05
		2023	17.21	14.16	18.82	1.04	6.02	1.9763	241.488	82	< 0.05
	Length/width ratio	2022	3.41	2.84	4.05	0.3	8.88	2.0569	181.7	82	< 0.05
		2023	3.23	2.61	3.7	0.28	8.63	2.0783	241.923	82	< 0.05
	Test weight (*kg*)	2022	650.57	567.5	704	30.44	4.68	2.0463	246.563	82	< 0.05
		2023	649.93	609	714.5	25.07	3.86	2.0202	246.301	82	< 0.05
	Specific gravity ($\text{g}*{\text{cm}}^{-3}$)	2022	1.2	1.1	1.31	0.03	2.39	1.8629	225.506	82	< 0.05
		2023	1.21	1.17	1.26	0.02	1.57	2.0206	11.599	248,82	< 0.05
	Degree of hardness	2022	-44.27	-56.6	-25.99	6.56	-14.82	1.9548	238.429	82	< 0.05
		2023	-47.04	-57.6	-26.91	6.39	-13.58	1.9328	236.498	82	< 0.05
Chemical quality	Fat content (%)	2022	6.9	4.45	9.3	1.06	15.32	2.0448	245.261	82	< 0.05
		2023	5.53	3.67	8.53	1.15	20.86	1.8655	242.63	82	< 0.05
	Protein content (%)	2022	18.44	15.63	22.37	1.48	8.03	2.0315	241.957	82	< 0.05
		2023	17.92	15.47	20.63	1.22	6.8	2.0513	239.17	82	< 0.05
	Total starch content (%)	2022	53.53	40.93	56.37	2.38	4.44	1.6293	241.375	82	< 0.05
		2023	53.21	50.8	56.43	1.2	2.25	2.0658	238.74	82	< 0.05
	Water content (%)	2022	8.66	7.83	9.2	0.22	2.49	1.9469	230.517	82	< 0.05
		2023	8.96	8.47	9.4	0.21	2.3	2.0233	237.617	82	< 0.05
	β-glucan content (%)	2022	2.87	2.17	4.22	0.4	14.03	2.0179	240.359	82	< 0.05
		2023	3	1.54	3.86	0.39	12.97	2.0242	239.676	82	< 0.05

#### Correlation.

**Fig 1:**
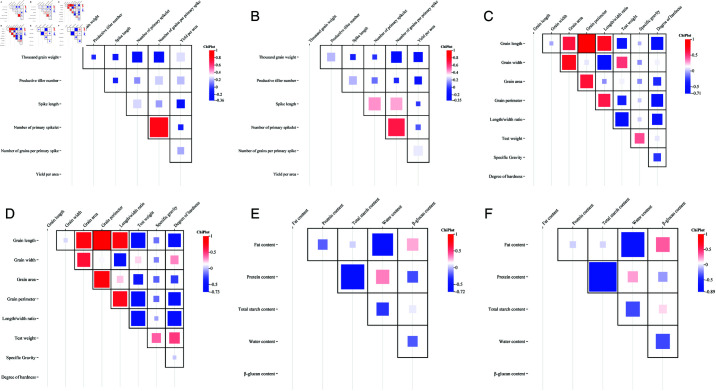
Correlations among different indices of agronomic and qualitative traits in 83 naked oat strains during 2022 and 2023. (**A**) Agronomic trait—2022. (**B**) Agronomic trait—2023. (**C**) Qualitative physical trait—2022. (**D**) Qualitative physical trait—2023. (**E**) Qualitative chemical trait—2022. (**F**) Qualitative chemical trait—2023.

In 2022, significant negative correlations were observed between TGW and PSS and between GS and TGW. There were significant positive correlations between SL and PSS and between GS and PSS. The GS was positively correlated with the PTP. YA positively correlated with TGW and negatively correlated with SL (Fig 1A, S2 Table). In 2023, significant positive correlations were observed between PSS and SL, GS and SL, and GS and PSS. There was a significant negative correlation between the GS and TGW. YA positively correlated with GS and negatively correlated with TGW (Fig 1B, S2 Table).

#### Key factor classification.

**Fig 2:**
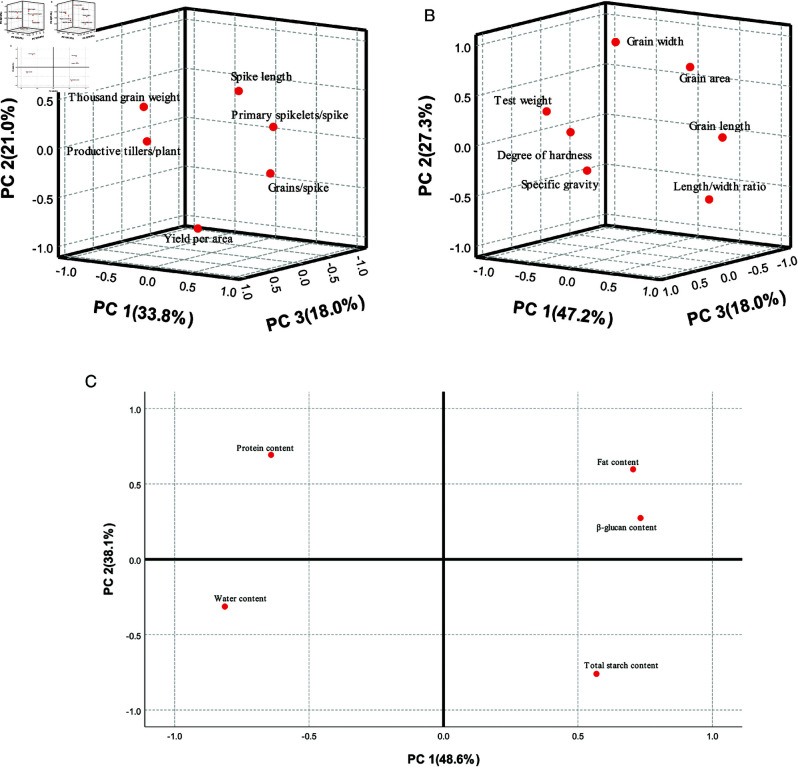
Principal component structure of variation in agronomic and qualitative traits for 83 naked oat strains during 2022 and 2023. (**A**) Agronomic trait. (**B**) Qualitative physical trait. (**C**) Qualitative chemical trait.

Multicollinearity was not detected among the traits (S2 Table). The average values of these six traits from 2022 and 2023 were suitable for PCA, as indicated by the Bartlett sphericity test (P < 0.05) and the KMO test (P = 0.458) [15] (Fig 2A). The eigenvalues of the first three principal components were greater than 1, with a cumulative contribution rate of 72.8%. The eigenvalue of PC1 was 2.027, which contributed to 33.8% of the total variance. Traits with high loadings on PC1 included GS (0.883), PSS (0.808), and TGW (–0.635), indicating that PC1 represents a yield factor. The vector relationship suggests that higher PSS and GS values correspond to lower TGW values. The eigenvalue of PC2 was 1.26, which contributed to 21.0% of the variance. Traits with high loading on PC2 included YA (–0.812) and SL (0.594), indicating that PC2 is related to spike length, with longer spikes corresponding to lower YA values. The eigenvalue of PC3 was 1.08, contributing to 18.0% of the total variance. The trait with the hightest loading on PC3 was PTP, suggesting that PC3 was a tiller factor.

#### Classification.

**Fig 3:**
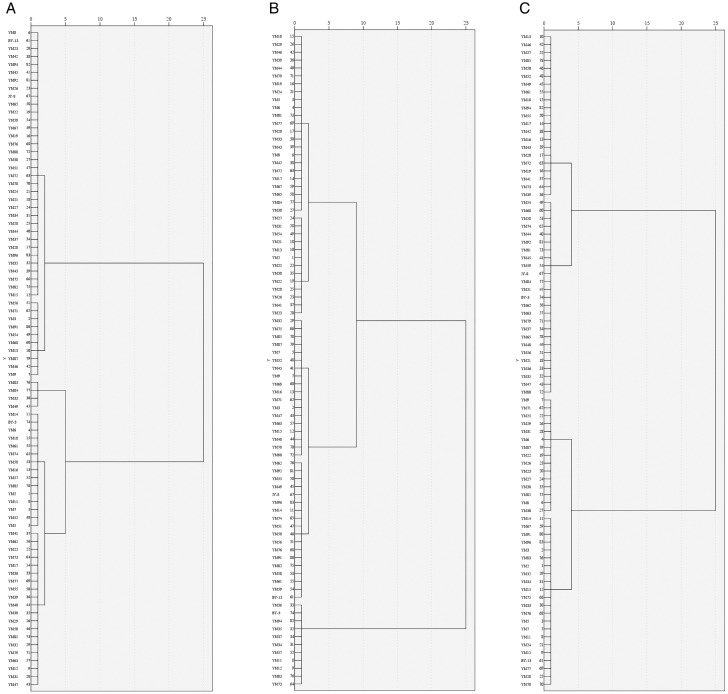
Cluster diagram of agronomic and qualitative traits for 83 naked oat strains during 2022 and 2023. (**A**) Agronomic trait. (**B**) Physically qualitative trait. (**C**) Chemically qualitative trait.

All strains from 2022 and 2023 were logically divided into three groups (Table 3 and S3 Table; Fig 3A).

**Table 3:** Biological characteristics, including the mean and coefficient of variation (CV) for agronomic and quality traits of 83 naked oat strains from 2022 to 2023, were analyzed using hierarchical clustering.

Traits			Group I	Group II	Group III	Group I	Group II	Group III
Agronomy	Thousand grain weight (*g*)	Mean	26.48	27.56	26.97	28.06	26.86	23.63
		CV (%)	12.47	8.97	9.09	7.35	11.43	10.62
	Productive tillers/plant	Mean	0.49	0.28	0.63	0.49	0.3	0.19
		CV (%)	72.39	91.5	85.04	76.42	78.05	125.16
	Spike length (*cm*)	Mean	15.54	14.16	17.79	14.75	14.92	15.59
		CV (%)	9.72	7.54	7.42	8.77	11.02	14.74
	Primary spikelets/spike	Mean	31.55	25.46	38.4	29.66	26.73	30.35
		CV (%)	13.38	14.36	14.6	19.65	17.14	15.06
	Grains/spike	Mean	69.56	55.68	87.3	67.01	58.71	59.91
		CV (%)	18.69	16.34	10.51	22.06	21.56	14.57
	Yield per area (*kg*/*ha*)	Mean	2.91	3.2	3.25	3.2	2.99	2.84
		CV (%)	17.2	13.25	4.36	12	16.42	21.11
Range of Score			0.416-0.585	0.179-0.405	0.677-0.728			
Physical quality	Grain length (*mm*)	Mean	7.61	7.15	6.4	7.51	7.16	6.42
		CV (%)	2.9	3.32	4.01	3.57	4.57	5.24
	Grain width (*mm*)	Mean	2.22	2.2	2.17	2.23	2.19	2.16
		CV (%)	4.6	5.78	4.91	4.72	5.52	5.53
	Grain area (${\text{mm}}^{2}$)	Mean	12.77	11.96	10.47	12.69	11.89	10.43
		CV (%)	4.49	5.69	5.38	4.9	5.84	5.53
	Grain perimeter (*mm*)	Mean	17.55	16.66	15.08	17.35	16.7	15.1
		CV (%)	2.2	2.75	3.39	2.9	3.83	4.28
	Length/width ratio	Mean	3.46	3.29	2.99	3.41	3.31	3.02
		CV (%)	6.37	8.13	7.32	6.88	8.56	9.37
	Test weight (*kg*)	Mean	640.45	654.92	667.07	643.38	652.72	671.5
		CV (%)	3.74	3.51	3.85	3.66	3.8	3.39
	Specific gravity ($\text{g}*{\text{cm}}^{-3}$)	Mean	1.21	1.21	1.21	1.2	1.21	1.21
		CV (%)	1.54	1.92	1.17	1.49	1.97	1.17
	Degree of hardness	Mean	-49.38	-45.39	-34.35	-48.29	-45.36	-35.53
		CV (%)	-5.59	-8.78	-13.44	-7.04	-10.91	-19.15
Range of Score			0.663-0.861	0.433-0.645	0.162-0.38			
Chemical quality	Fat content (%)	Mean	5.29	7.26	6.72	6.31	6	6.51
		CV (%)	12.96	7.31	6.5	17.83	16.12	11.47
	Protein content (%)	Mean	18.28	17.6	18.52	17.78	18.36	19.3
		CV (%)	4.84	6.98	6.51	5.08	6.16	5.8
	Total starch content (%)	Mean	53.37	54.01	52.85	53.87	53.15	51.95
		CV (%)	1.81	1.96	4.1	1.6	3.02	4.26
	Water content (%)	Mean	8.97	8.6	8.75	8.78	8.85	8.85
		CV (%)	1.26	1.55	1.17	2.39	2.07	1.38
	β-glucan content (%)	Mean	2.74	3.22	2.99	2.98	2.84	3.02
		CV (%)	9.56	6.8	4.92	10.57	9.57	7.23
Range of Score			0.08-0.375	0.53-0.813	0.396-0.512	0.575-0.791	0.415-0.57	0.293-0.380

Group I contained 35 strains, including BY-3, which accounted for 42.2% of the total number of strains (*D* values = 0.416-0.585). The main characteristics of Group I were as follows: (1) the lowest values for TGW and YA, (2) the highest CV values for TGW, YA, SL, and GS, and (3) the lowest CV values for PTP and PSS. This group consisted of medium-yield strains. Group II contained 44 strains, including BY-13 and JY-8, accounting for 53.0% of all strains (*D* values = 0.179-0.405). Its main features were as follows: (1) the highest value for TGW, (2) the lowest values for PTP, PSS, and GS, (3) the lowest CV value for TGW, and (4) the highest CV value for PTP. This group represented low-yield strains. Group III contained four strains, accounting for 4.8% of all strains (*D* values = 0.677-0.728). Its main characteristics were as follows: (1) the highest values for PTP, SL, PSS, GS, and YA, (2) the lowest CV values for SL, GS, and YA, and (3) the highest CV value for PSS. This group comprised of high-yield strains. Strain YM49 had the highest comprehensive score with a *D* value of 0.73, a high PTP of 1.4, and was ranked among the top strains for SL, GS, and PSS, whereas TGW and YA were in the middle range of all strains.

### Physically qualitative traits

#### Variability and genetic diversity.

Significant differences were observed between the 83 strains for each trait (Table 2). The average values of GL, GW, GA, GP, and SG in 2023 were lower than those in 2022. GA showed the largest increase from 2022 to 2023, whereas SG exhibited the smallest increase. In 2022, the CV of the eight traits ranged from 2.4% to 14.8% in the following order from highest to lowest: HD > GA > L/W > GL > GW > GP > TW > SG. The highest CV was observed for HD. The strain with the highest CV for this trait was YM38, whereas YM37 had the lowest CV. SG had the lowest CV, with YM77 showing the highest and YM49 showing the lowest CV for this trait. The genetic diversity indices for these eight traits ranged from 1.8629 to 2.0718, with the order from largest to smallest being GA > L/W > TW > GW > GL > HD > GP > SG. In 2023, the CV for these eight traits ranged from 1.6% to 13.6%, with the traits ordered from highest to lowest CV as follows: HD > GA > L/W > GL > GP > GW > TW > SG. HD had the highest CV, with YM77 showing the highest CV for this trait, whereas YM37 had the lowest CV. SG had the lowest CV, with YM74 showing the highest CV for this trait and YM55 showing the lowest. The genetic diversity indices for these traits ranged from 1.9328 to 2.0783, with the order from the largest to smallest being L/W > GA > GW > SG > TW > GL > GP > HD.

#### Correlation.

In 2022, significant negative correlations are observed between L/W and GW, TW and GL, TW and GP, TW and L/W, SG and TW, HD and GL, HD and GA, are observed HD and GP, and HD and L/W. Significant positive correlations are observed between GA and GL, GA and GW, GP and GL, GP and GA, L/W and GL and L/W and GP. HD negatively correlated with SG, and TW positively correlated with GW (Fig 1C, S2 Table). In 2023, significantly negative correlations are observed between L/W and GW, TW and GL, TW and GA, between TW and GP, TW and L/W, SG and TW, HD and GL, HD and GP, and HD and L/W. Significantly positive correlations are observed between GA and GL, GA and GW, GP and GL, GP and GA, L/W and GL, L/W and GP, HD and GW, and HD and TW. L/W positively correlated with GA, and HD negatively correlated with GA (Fig 1D, S2 Table).

#### Key factor classification.

The correlation analysis revealed a strong relationship between GL and GP (S2 Table). To minimize multicollinearity in the PCA, GP was excluded from subsequent analysis. The average values of these seven traits from 2022 to 2023 were suitable for PCA, as indicated by the Bartlett sphericity test (P < 0.05) and the KMO test (P = 0.571) (Fig 2B). The eigenvalues of the first three principal components were all greater than 1, with a cumulative contribution rate of 92.5%. The eigenvalue of PC1 was 3.301, accounting for 47.2%. Traits with high loadings were GL (0.937), L/W (0.918), HD (-0.792) and TW (-0.771). The vector relationship suggests that as the GL and L/W increased, the TW and HD decreased. The eigenvalue of PC2 was 1.91, which contributed 27.3%. The traits with high loadings were GW (0.913) and GA (0.876), indicating that PC2 was a grain-size factor. The vector relationship indicates that as the grain width (GW) increased, the grain area(GA) also increased. The eigenvalue of PC3 was 1.26, which contributed 18.0%. Traits with high loadings were SG (0.919) and TW (0.506), making PC3 a grain saturation factor. The vector relationship suggested that as SG increased, TW also increased.

#### Classification.

Based on eight traits evaluated in 2022 and 2023, all strains were divided into three groups (Table 3 and S3 Table; Fig 3B). Group I consisted of 36 strains, accounting for 43.4% of all the strains (*D* values = 0.663–0.861). The main characteristics of Group I were as follows: (1) highest values for GL, GW, GA, GP, L/W, and SG; (2) lowest values for TW and HD; and (3) lowest coefficient of variation (CV) for GL, GW, GA, GP, L/W, and HD. Most of the strains in this group had large grains. Group II also contained 36 strains, including BY-13 and JY-8, accounting for 43.4% of all the strains (*D* values = 0433–0.645). The main features of Group II were as follows: (1) lowest SG value; (2) highest CV for GW, GA, L/W, and SG; and (3) lowest CV for TW. Most of the strains in this group had medium-sized grains. Group III comprised 11 strains, including BY-3, accounting for 13.3% of all strains (*D* values = 0.162–0.38). The key characteristics of Group III were as follows: (1) lowest values for GL, GW, GA, GP, and L/W; (2) highest values for TW and HD; and (3) highest CV for GL, GP, TW, and HD. The strains in this group had small, hard, and full grains. Strain YM23 had the highest comprehensive score (*D* value = 0.861), with a maximum GA of $13.83\;{\text{mm}}^{2}$ and a maximum GP of 18.23 *mm*. The GL and GW values of YM23 were among the highest across all strains, while GL, L/W, TW, and SG were in the middle range.

### Chemical quality traits

#### Variability and genetic diversity.

Significant differences were observed between the 83 strains for each trait (Table 2). The average values of β-gC and WC in 2023 were higher than those in 2022 (Table 2). In 2022, the CV values of five traits ranged from 2.5% to 15.3%, with the order of CV from largest to smallest being FC > β-gC > PC > TSC > WC. The average FC was 6.9%. The highest FC value was observed in strain YM39, whereas the lowest was observed in strain YM82. The average WC was 8.7%, and the CV was the smallest (7.8%-9.2%). The highest WC was observed in strain YM83, whereas the lowest was observed in YM39. The genetic diversity indices for these five chemical quality traits ranged from 1.6293 to 2.0448, with the order from the largest to smallest being FC > PC > β-gC > WC > TSC. In 2023, the CV values of these five traits ranged from 2.3% to 20.9%, with the following order of CV from largest to smallest: FC > β-gC > PC > WC > TSC. The average FC level was 5.5%, with values ranging from 3.7% to 8.5%, and the CV value for FC was the highest (20.9%) of the five traits. The highest FC value was found in strain YM52 and the lowest in strain YM71. The average TSC was 53.2%, ranging from 50.8% to 56.4%, and the CV value for TSC was the lowest among the five traits. The strains with the highest and lowest TSC were YM62 and YM68, respectively. The genetic diversity index for these traits ranged from 1.8655 to 2.0658, with the order of genetic diversity from the largest to smallest being TSC > PC > β-gC > WC > FC.

#### Correlation.

In 2022, a significant positive correlation was observed between WC (water content) and PC (protein content). There were significant negative correlations between TSC (total start content) and PC, WC and FC (fat content), and WC and TSC. β-gC (β-glucan) positively correlated with FC, and negatively correlated with PC (Fig 1E, S2 Table). In 2023, significant negative correlations was noted between TSC and PC, WC and FC, WC and TSC, β-gC and FC, and β-gC and WC (Fig 1F, S2 Table).

#### Key factor classification.

Multicollinearity was not detected among the traits (S2 Table). The data for the five aforementioned chemical quality traits for 2022 and 2023 were suitable for PCA (Bartlett’s sphericity test, P < 0.05, and the KMO test, P = 0.555) (Fig 2C). The eigenvalues of the first two principal components were both greater than 1, and their cumulative contribution rate was 80.4%. The eigenvalue of PC1 was 2.431, with a contribution rate of 48.6%. The traits with high loadings were WC (-0.813), β-gC (0.733), FC (0.705), PC (-0.642), and TSC (0.569). The vector relationship indicates that the lower the WC, the higher the β-gC, FC, and TSC, and the lower the PC. The eigenvalue of PC2 was 1.587, with a contribution rate of 31.7%. The traits with high loadings were TSC (-0.76), PC (0.693), and FC (0.597). The vector relationship showed that the lower the TSC, the higher the PC and FC.

#### Classification.

All strains tested in 2022 and 2023 were divided into three groups (Table 3 and S3 Table; Fig 3C). Group I included 37 strains, including BY-13, accounting for 44.6% of all strains (*D* values = 0.08-0.375). The main features of Group I were: (1) highest WC value, (2) lowest FC and β-gC values, (3) highest CV levels for FC and β-gC, and (4) lowest CV levels for PC and TSC, indicating low nutritional quality. Group II included 21 strains, accounting for 25.3% of all the strains (*D* values = 0.530-0.813). The main features of Group II were: (1) highest FC, TSC, and β-gC values, and (2) lowest PC and WC values, with the highest CV. Most strains in this group had high β-glucan levels. Group III contained 25 strains, including BY-3 and JY-8, accounting for 30.1% of all the strains (*D* values = 0.396-0.512). The main features of Group III were: (1) highest PC level, (2) lowest TSC level, with the highest CV, and (3) lowest CV values for FC, WC, and β-gC. Most strains in this group possessed high protein content. Strain YM39 had the highest comprehensive score with a *D* value of 0.813 and an FC value of 8.45. As per protein, starch, and β-glucan levels, Group III ranked first among all strains.

### Status of 19 traits of 83 strains across mean of both 2022 and 2023

#### Correlation.

**Fig 4:**
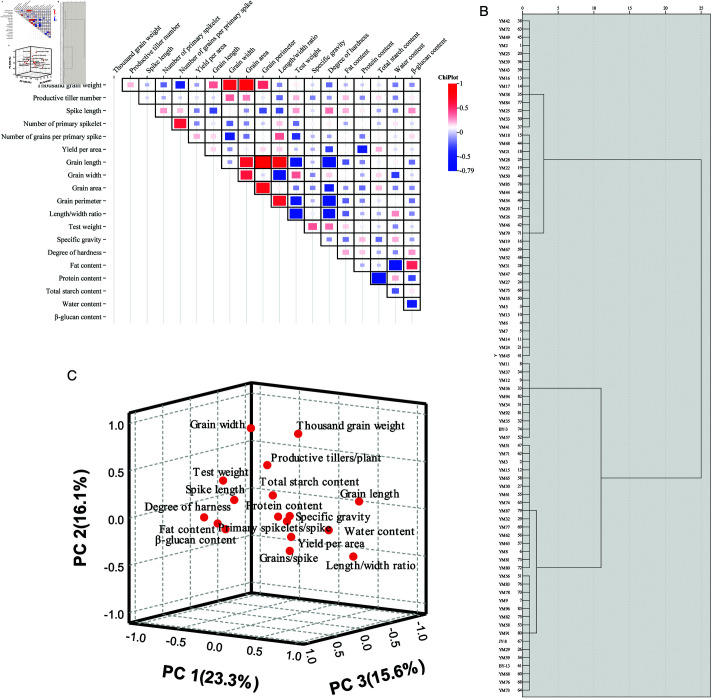
Correlations (A), principal component structure (B), and cluster diagram (C) of the 2-year average agronomic and qualitative traits for 83 naked oat strains.

Significant positive correlations were observed between GA and GL, GA and GW, GA and PTP, GA and TGW, GL and TGW, GP and GA, GP and GL, GP and TGW, GW and PTP, GW and TGW, HD and SL, HD and TW, L/W and GL, L/W and GP, L/W and GS, GS and PSS, GS and TGW, TW and GW, TW and SG, WC and L/W, and β-gC and FC. There were the significant negative correlations between GL and SL, GP and SL, GW and GS, HD and GA, HD and GL, HD and GP, HD and L/W, HD and YA, L/W and GW, L/W and SL, PC and YA, TSC and PC, TSC and SG, TW and GL, TW and GP, TW and L/W, TW and GS, WCand FC, WC and GW, β-gC and PC, and β-gC and WC. FC positively correlated with HD and SL and negatively correlated with GP, L/W and GL. GW negatively correlated with PSS. HD positively correlated with TGW. L/W negatively correlated with PTP. GS positively correlated with YA. PSS positively correlated with SL and negatively correlated with TGW. TSC positively correlated with YA. TW positively correlated with SL. WC positively correlated with PC and SG and negatively correlated with TSC. β-gC positively correlated with HD and SL (Fig 4A, S4 Table).

#### Key factor classification.

Correlation analysis revealed two strong relationships either between TGW and GA or between GL and GP (S4 Table). To minimize multicollinearity in the PCA, GA and GP were excluded from subsequent analyses. The data for all 17 traits of the 83 strains for the averages of 2022 and 2023 were suitable for PCA (Bartlett sphericity test, P < 0.05; KMO test, P = 0.560) (Fig 4B). The eigenvalues of the first six principal components were greater than 1, and their cumulative contribution rate was 78.7%. The eigenvalue of PC1 was 3.967 and the contribution rate was 23.3%. The traits with high loadings were GL (–0.730), WC (–0.504), L/W (–0.933), HD (0.652), TW (0.725), and GW (0.657). Six traits were related to the qualitative physical and chemical features. The vector relation indicated that the larger the grain, the softer and less full it was. The eigenvalue of PC2 was 2.738, and its contribution rate was 16.1%. The traits with high loadings were PC (–0.798), TSC (0.693) and WC (–0.514). These traits are related to the qulitative chemical traits. The vector relation was as follows: the higher the TSC, the lower the GS, PC, and WC. The eigenvalue of PC3 was 2.645 and its contribution rate was 15.6%. The traits with high loadings were GS (0.718), TGW (–0.709), GW (–0.506), β-gC (0.563), and PSS (0.507). These traits correlated with all the other traits. The vector relationship was that the lower the TGW and GA values, the higher the GS, β-gC, GS and PSS values. The eigenvalue of PC4, PC5 and PC6 were 1.51, 1.31 and 1.21, respectively. The contribution rates of PC4, PC5 and PC6 were 8.9%, 7.7%, and 7.1%, respectively. The traits with the high loading for PC4, PC5 and PC6 were YA (–0.448), PSS (0.609), and SG (0.721), respectively.

#### Classification.

On the basis of the average values for all 19 traits of 2022 and 2023, all strains were divided into three groups (Table 3 and S3 Table; Fig 4C).

Group I contained 42 strains, accounting for approximately 50.6% of all the strains (*D* values = 0.575–0.791). The first group of main features of Group I were: (1) highest PTP, GS and YA values, (2) lowest SL value, (3) highest CV for PSS and GS, and (4) lowest CV values for TGW, PTP, SL and YA, suggesting high yield. The second group of main features was: (1) highest GL, GW, GA, GP, L/W and HD values with the lowest CV value, and (2) lowest TW and SG values, suggesting large grain. The third group of features included: (1) highest TSC value, and (2) lowest PC and WC values, and (3) highest CV’s for FC, WC and β-gC, and (4) lowest CV values for PC and TSC, suggesting grain with a low nutrient level. Group I consisted of high-yield, large-grain and low-nutrient strains.

Group II contained 31 strains, including BY-13 and JY-8, which accounted for 37.4% of all the strains (*D* values = 0.415–0.57). The first group of main features of Group II were (1) highest TGW with the highest CV, and (2) lowest PSS and GS, suggesting strains with medium yields. The second group of main features included: (1) highest SG value, and (2) highest CV values for GA, TW, and SG, suggesting medium grain size. The third Group of features were: (1) lowest FC and β-gC levels, (2) highest CV for PC, suggesting low nutrition. Group II comprised a collection of medium-yield, low-nutrient, and medium-sized grain strains.

Group III consisted of 10 strains, including BY-3, which accounted for 12.1% of all strains (*D* values = 0.293–0.380). The first group of main features of Group III were: (1) highest SL and PSS values, (2) lowest TGW, PTP and YA values, (3) highest CV for PTP, SL, GS and YA, and (4) lowest CV for PSS, suggesting a low yield. The second group of main features included: (1) highest TW value, and (2) lowest GL, GW, GA, GP, L/W and HD values, and (3) highest CV for GL, GW, GP, L/W and HD, and (4) highest CV for TW and SG, suggesting a small grain. The third group features were: (1) highest FC, PC, WC, and β-gC values, (2) lowest CV for FC, WC and β-gC, and (3) lowest TSC value, with the highest CV, suggesting a high nutrient group. Group III consisted of small-grain strains with low yield and high nutrition content. Strain YM41 showed the highest comprehensive score, with a *D* value of 0.79 and the largest YA. The TGW, PTP, SL, GS, FC, TSC, WC, β-gC, GL, GW, GA, GP, and L/W values in all strains were remarkable. The PSS, TW and HD values were intermediate for all strains.

#### Variety screening.

Grey relational analysis was applied to evaluate trait importance and identify high-performing oat strains based on data from 2022 and 2023, and their two-year averages. Across all three datasets, PTP consistently exhibited the highest weight coefficient (0.220 in 2022, 0.345 in 2023, and 0.168 for the average), highlighting its pivotal role in overall performance assessment. Additional traits with relatively high weight contributions included β-gC, FC, TW, HD, and L/W; however, their rankings varied slightly between the years (Table 4). Based on the weighted degrees of correlation, several strains were repeatedly ranked among the top performers. Strain YM49 demonstrated consistently strong performance across all evaluations (r = 0.482–0.638), followed by YM47, YM41, YM84, and YM48 (Table 5). These strains exhibited stable and balanced trait profiles, making them promising candidates for breeding programs. This integrative, multi-year assessment underscores the robustness of grey relational analysis in identifying key yield- and quality-related traits and provides a scientific basis for the targeted selection of elite germplasm for naked oat improvement.

**Table 4:** Correlation coefficients of agronomic and quality traits for 83 naked oat strains from 2022 to 2023.

Traits		2022			2023			2022-2023		
		Correlation degree	Weight coefficient/ωı	Rank	Correlation degree	Weight coefficient/ωı	Rank	Correlation degree	Weight coefficient/ωı	Rank
Agronomy	Thousand grain weight (*g*)	0.98	0.039	11	0.987	0.019	16	0.983	0.032	14
	Productive tillers/plant	0.885	0.222	1	0.759	0.345	1	0.914	0.168	1
	Spike length (*cm*)	0.983	0.033	14	0.961	0.056	5	0.975	0.048	9
	Primary spikelets/spike	0.974	0.05	8	0.971	0.042	8	0.971	0.056	5
	Grains/spike	0.97	0.057	6	0.979	0.03	12	0.982	0.036	12
	Yield per area (*kg*/*ha*)	0.982	0.035	12	0.984	0.023	15	0.986	0.027	17
Physical quality	Grain length (*mm*)	0.977	0.045	10	0.948	0.074	2	0.959	0.081	3
	Grain width (*mm*)	0.966	0.065	4	0.968	0.046	6	0.974	0.051	8
	Grain area (${\text{mm}}^{2}$)	0.994	0.012	19	0.969	0.044	7	0.993	0.014	19
	Grain perimeter (*mm*)	0.991	0.017	18	0.978	0.031	11	0.989	0.022	18
	Length/width ratio	0.982	0.034	13	0.956	0.063	3	0.973	0.053	7
	Test weight (*kg*)	0.971	0.056	7	0.988	0.018	17	0.981	0.037	11
	Specific gravity ($\text{g}*{\text{cm}}^{-3}$)	0.976	0.047	9	0.99	0.014	18	0.984	0.032	15
	Degree of hardness	0.965	0.068	3	0.972	0.039	9	0.969	0.06	4
Chemical quality	Fat content (%)	0.989	0.021	17	0.974	0.038	10	0.978	0.043	10
	Protein content (%)	0.961	0.075	2	0.99	0.014	19	0.971	0.056	6
	Total starch content (%)	0.969	0.06	5	0.96	0.057	4	0.94	0.118	2
	Water content (%)	0.984	0.031	15	0.982	0.026	13	0.984	0.031	16
	β-glucan content (%)	0.984	0.03	16	0.984	0.023	14	0.982	0.035	13

**Table 5:** Absolute correlation values of 83 naked oat strains from 2022 to 2023 based on weighted correlation analysis.

Name of germplasm	2022		2023		2022-2023	
	Weighted correlation degree	Weighted order	Weighted correlation degree	Weighted order	Weighted correlation degree	Weighted order
YM2	0.468	13	0.315	41	0.48	18
YM3	0.405	39	0.271	72	0.366	74
YM5	0.436	24	0.389	26	0.463	26
YM6	0.493	6	0.427	21	0.498	11
YM7	0.379	54	0.514	12	0.449	34
YM8	0.358	64	0.278	68	0.362	77
YM9	0.336	75	0.213	83	0.286	83
YM11	0.362	62	0.299	49	0.403	59
YM12	0.302	82	0.288	57	0.336	80
YM13	0.457	19	0.347	36	0.47	23
YM14	0.525	3	0.262	77	0.438	41
YM15	0.468	12	0.301	47	0.457	30
YM16	0.421	32	0.293	56	0.426	49
YM17	0.52	4	0.303	45	0.48	17
YM18	0.51	5	0.3	48	0.482	16
YM19	0.38	53	0.279	67	0.386	62
YM20	0.382	50	0.328	38	0.445	37
YM21	0.418	34	0.28	65	0.427	47
YM22	0.392	45	0.273	71	0.378	68
YM23	0.381	52	0.314	42	0.386	63
YM24	0.325	78	0.255	79	0.331	82
YM25	0.464	15	0.296	52	0.434	43
YM26	0.339	73	0.282	64	0.364	76
YM27	0.332	76	0.271	73	0.339	79
YM28	0.371	56	0.282	63	0.382	65
YM29	0.378	55	0.283	61	0.373	72
YM30	0.385	48	0.28	66	0.37	73
YM31	0.423	29	0.306	44	0.405	58
YM32	0.416	36	0.32	40	0.42	52
YM33	0.463	16	0.358	32	0.489	15
YM34	0.311	81	0.519	10	0.469	24
YM35	0.331	77	0.335	37	0.432	44
YM36	0.324	79	0.532	8	0.508	10
YM37	0.368	60	0.364	30	0.476	20
YM38	0.438	23	0.35	34	0.446	35
YM39	0.46	17	0.566	4	0.547	5
YM41	0.656	1	0.273	70	0.535	6
YM42	0.42	33	0.285	60	0.414	55
YM43	0.399	42	0.517	11	0.476	21
YM44	0.443	22	0.301	46	0.426	48
YM45	0.394	44	0.436	19	0.454	33
YM46	0.413	37	0.526	9	0.478	19
YM47	0.551	2	0.475	16	0.575	2
YM48	0.47	11	0.553	5	0.55	4
YM49	0.49	7	0.638	2	0.614	1
YM50	0.455	20	0.416	22	0.494	13
YM51	0.37	58	0.294	53	0.386	64
YM52	0.413	38	0.376	27	0.491	14
YM54	0.349	68	0.546	7	0.46	28
YM55	0.473	10	0.234	82	0.413	56
YM56	0.322	80	0.308	43	0.416	53
YM57	0.34	72	0.405	25	0.457	29
YM58	0.357	65	0.549	6	0.509	9
YM59	0.349	69	0.286	58	0.374	71
YM61	0.35	66	0.413	23	0.431	46
YM62	0.425	27	0.274	69	0.416	54
YM63	0.37	57	0.365	29	0.461	27
YM65	0.423	28	0.361	31	0.445	38
YM67	0.482	8	0.321	39	0.437	42
YM68	0.482	9	0.245	81	0.423	50
BY-13	0.337	74	0.27	74	0.332	81
YM71	0.349	67	0.454	17	0.38	66
YM72	0.458	18	0.286	59	0.44	40
YM73	0.368	59	0.427	20	0.475	22
YM74	0.428	25	0.357	33	0.456	32
YM75	0.384	49	0.484	13	0.457	31
JY-8	0.449	21	0.299	50	0.431	45
YM76	0.405	40	0.253	80	0.365	75
YM77	0.416	35	0.294	54	0.422	51
YM78	0.389	47	0.259	78	0.375	70
YM79	0.466	14	0.48	15	0.534	7
YM80	0.423	30	0.282	62	0.406	57
YM81	0.361	63	0.677	1	0.518	8
BY-3	0.389	46	0.294	55	0.388	61
YM82	0.422	31	0.373	28	0.445	39
YM83	0.363	61	0.481	14	0.469	25
YM84	0.426	26	0.573	3	0.55	3
YM85	0.398	43	0.447	18	0.494	12
YM87	0.343	70	0.267	76	0.341	78
YM91	0.382	51	0.27	75	0.379	67
YM92	0.34	71	0.35	35	0.4	60
YM94	0.29	83	0.298	51	0.376	69
YM96	0.404	41	0.413	24	0.445	36

## Discussion

We measured 19 agronomic and qualitative traits in 83 naked oat strains over two consecutive years at the same agricultural location. The results indicated that significant differences were observed among the strains for each trait each year, and the average values of two agronomic traits (TGW, PSS), five physical quality traits (GL, GW, GA, GP, SG), and two chemical quality traits (WC and β-gC) were higher in 2023 than in 2022, which may be attributed to the high rainfall during the grain filling period in July 2022 [37]. CV values greater than 10% suggest significant differences in traits between the strains [38]. The CV values for six agronomic, one physical quality (HD), and two chemical quality traits (FC and β-gC) were above 10%, reflecting considerable differences and variation in these traits among the 83 strains. This study provides a rich genetic foundation and valuable parent material for the development of new naked oat varieties. The CV values for FC and β-gC were higher than for TSC, consistent with previous findings [39,40]. Notable differences in genetic diversity were observed between the two years for traits such as YA, SG, TW, HD, FC, and TSC. Phenotypic identification and trait evaluation are significantly influenced by environmental and cultivation factors, highlighting the limitations of relying on data from a single year [41]. The genetic diversity indices for GS and TGW were notably high, supporting the findings of Zhang *et al*. [42]. The rankings of CV values and genetic diversity indices did not align, a result that was also reported by Liang *et al*. [43]. Traits such as SL, PSS, GS, TGW, GL, GP, GA, and TW exhibited stable heritability with high CV and diversity indices. These findings suggest that GS, FC, and β-gC have strong genetic bases for selection and show significant potential for strain improvement.

Among agronomic traits, PTP, PSS, TGW, and GS are crucial for the yield of naked oats [44,45]. We found a negative correlation between TGW and GS and a positive correlation between GS and PSS and between PSS and SL, as observed by Nan *et al*. [46] and Shi *et al*. [32]. These correlations indicate that longer spike length (SL) is associated with greater numbers of PSS and GS but with a reduction in TGW. This pattern suggests a tradeoff between grain number and grain size, a phenomenon commonly observed in cereal crops [47]. Notably, our findings support the concept of coarse- and fine-tuning of grain yield, wherein GS acts as a coarse-tuning factor with a dominant influence on yield, whereas TGW contributes as a fine-tuning factor. Such hierarchical contribution has also been reported in emmer wheat and sesame plants under different stress conditions [48,49]. Therefore, in various screening and breeding strategies, priority should be given to enhancing GS and PSS to maximize yield potential, while managing TGW to ensure adequate grain size and processing quality. Balanced selection that incorporates SL, PSS, GS, and TGW can facilitate the development of high-yield oat varieties with stable agronomic performance and quality. Regarding physical quality, GL was negatively correlated with TW and HD, whereas GP was positively correlated with L/W but negatively correlated with TW and HD. TW was positively correlated with SG and negatively correlated with L/W. These findings were consistent with previous reports [50,51]. The correlation between the TW and grain size is crucial for breeding. To improve TW, strains with small L/W ratios should be bred to meet processing requirements, as TW can be predicted from L/W. In terms of qualitative chemical traits, PC and TSC were negatively correlated, whereas β-gC and FC showed a positive correlation, consistent with the findings of Zhang *et al*. [52]. The correlations between the two-year average values of all traits revealed that YA was positively correlated with both GS and TSC but negatively correlated with HD and PC. L/W was positively correlated with GS, GL, and GP but negatively correlated with PTP, GW, TW, HD, and FC. FC was positively correlated with SL, HD, and β-gC but negatively correlated with GL, GP, and L/W. β-gC was positively correlated with SL, HD, and FC, but negatively correlated with PC. In oat germplasm resources, agronomic traits are often negatively correlated with quality traits [53,54], although some studies show no significant correlations between them [55,56]. Protein content (PC) is the key factor reflecting the nutritional quality of oats and is related to PTP and PSS [57]. The correlation between YA and FC has been reported to be significant [46]; however, this was not the case in our study or in other reports [58,59]. These findings suggest that the relationship between agronomic and quality traits should be balanced when screening oat strains. Traits such as YA, β-gC, L/W, TW, PC, and FC should be considered when evaluating oat varieties focused on grain production. Increasing YA, FC, and β-gC, while reducing L/W, is more important for strain improvement than focusing on any single trait. The degree of hardness (HD) not only improves yield per area (YA) but also enhances quality by positively affecting the L/W, TW, FC, and β-gC. Increasing GS contributes to a higher yield and enhances L/W and TW, which are two key factors in physical quality. However, the negative correlation between PC and both YA and β-gC highlight the inherent tradeoff between high yield and superior nutritional quality in oat breeding. Strains with higher yields tend to have lower protein content, whereas those with superior nutritional profiles tend to show reduced yield stability. Strategies such as hybridization and advanced breeding techniques such as genomic selection can help improve yield and nutritional quality simultaneously. Critical traits, such as effective tillering, and protein content, should be prioritized in breeding programs to optimize both yield and quality. The considerable genetic diversity observed in these traits offers an opportunity to develop varieties that excel in both yield and nutritional value, which are essential for advancing functional food production. Future breeding efforts should emphasize multi-trait selection and use genomic tools to strike a balance between yield and quality, benefiting both producers and consumers.

To effectively predict the yield and guide breeding decisions, we analyzed the loading patterns of traits across the principal components (PCs) and examined their relevance to specific breeding goals [60–62]. In PC1, which captured the largest proportion of the phenotypic variance, PSS, GS, and TGW showed the highest loadings. This indicates that PC1 predominantly represents yield-related traits, with GS contributing the most significantly to yield. The inverse relationship between GS and TGW suggests a tradeoff between grain number and grain size, with higher grain numbers potentially leading to smaller grains. Consequently, breeding programs targeting yield improvement should prioritize GS and PSS, while maintaining TGW within an optimal range to balance grain size and yield. PC2, which captured the second largest variance, had high loadings for traits related to plant architecture and yield distribution, such as YA and SL. Although these traits were less influential than the yield components in PC1, they were important for optimizing plant structure and maximizing yield potential. In breeding programs, these traits can help balance overall plant architecture, ensuring that yield is not compromised by excessive elongation of spikes or an unbalanced tillering structure. PC3 was strongly influenced by PTP, a trait related to tillering capacity that plays a key role in determining the number of productive tillers. PTP should be maintained within an optimal range to ensure efficient resource allocation and maximize productive tillers. Excessive tillering could lead to resource dilution, whereas inadequate tillering could limit yield potential. In terms of grain quality, PC1 played a significant role by capturing traits such as GL, WC, and L/W, which are related to grain size and texture. PC2 further reflected the variation in grain shape (L/W) and appearance (GA), which are critical for processing quality. On the other hand, PC3 was linked to traits such as HD and TW, which indicate grain fullness and density. These traits are crucial for determining the overall grain quality and should be prioritized in breeding programs aimed at improving processing quality and consumer acceptability. Regarding chemical quality, PC2 provided insights into traits such as PC, TSC, and FC, which are important for nutritional content. These traits were largely independent of yield-related traits, suggesting that improvements in chemical composition can be pursued without significantly compromising the yield. The relative independence of these traits offers breeders the opportunity to simultaneously enhance yield and quality . Overall, the PCA of 19 traits across 83 naked oat strains revealed that the first three PCs accounted for over 55% of the total phenotypic variance. These components represented the key aspects of yield (PC1), chemical composition (PC2), and structural traits (PC3). The observed tradeoffs, such as the inverse relationship between GS and TGW, emphasize the need for integrated breeding strategies that balance yield and quality. The additional variance explained by PCs 4–6 underscores the importance of agronomic and appearance traits such as SG and YA, which should be incorporated into selection indices. In conclusion, to optimize both yield and quality in naked oats, breeders should focus on traits that influence grain size and weight, particularly GS, which has a direct effect on yield. Simultaneously, nutritional factors (such as PC, β-gC, and FC) should be selected independently, allowing for improvements in both yield and quality. Parent material selection should be guided by the principal component scores, prioritizing strains with desirable traits and supporting characteristics that enhance overall performance.

In terms of agronomic quality, Group I strains exhibited medium yield with considerable variation in SL and GS, making them ideal parent candidates for the breeding of specific panicle varieties. Group II consisted of low-yield strains. However, given that tillering is a crucial agronomic trait that influences oat yield and that the number of tillers affects root development, Group II strains could serve as suitable parents for breeding lodging-resistant varieties. Group III strains, which are high-yield varieties, are excellent parents for the development of new high-yield varieties. YM49, a high-yield strain, is a remarkable resource for germplasm innovation aimed at increasing yield. Regarding the physical quality, three groups were identified based on grain size: (I) large, (II) medium, and (III) small grains. Group I included superior varieties of large-grain oats that offered opportunities for selection. In Group III, the strains with the lowest L/W ratios could be used as parents for propagating new varieties with broader grain widths (GW). YM23, a Group III member, is a top-ranked variety that can serve as a crossing parent for strains used for the production of oatmeal or other varieties that require large grains. In terms of chemical quality, the strains were grouped according to their protein content, β-glucan content, or lower nutritional value. YM39 demonstrated outstanding performance, and could be a key resource for breeding programs aimed at enhancing the nutritional value of oat strains. The summary, the 83 strains investigated in this study were categorized into three groups. Group I consisted of high-yield, large-grain, low-nutrition strains with high GS and YA values, rendering them suitable for breeding hybrid parents of high-yield, large-grain varieties. Group II included the intermediate-yield, medium-grain, and low-nutrition strains with high TGW and SG values. The high TGW of the Group II strains could be leveraged to breed hybrid parents aimed at increasing TGW. Group III comprised high-yield, small-grain, high-nutrition strains. These strains, characterized by high L/W, TW, FC, and β-gC could serve as hybrid parents to enhance both grain nutrition and size. YM41 stands out as a high-yield and high-quality variety with potential utility in production trials. BY-13, a non-high-yield, medium-sized, low-nutrition strain; JY-8, a non-high-yield, medium-sized, low-nutrition strain; and BY-3, a non-high-yield, non-large-sized, high-protein strain fell within the non-high-level group and showed substantial potential for improvement. These genetically diverse varieties, with large-grains, high-yields, and high-quality, provide valuable material for the breeding of naked oats. Several other strains present opportunities as backup varieties for further development and eventual production applications.

We identified YM49 as the only strain that consistently achieved high comprehensive evaluation scores in both 2022 and 2023, indicating its high degree of stability across different growing seasons. Such stability implies a strong adaptability to varying environmental conditions, which is a highly desirable trait in breeding programs intended for reliable performance across years and locations. In the context of increasing climate variability, the development of cultivars with stable phenotypic expression under fluctuating environmental conditions is essential to ensuring sustainable agricultural productivity. The considerable differences in performance observed among the many top-performing strains between the two years were likely attributable to year-to-year climatic variation. Therefore, the consistent performance of YM49 not only emphasizes its value as a stable germplasm resource but also highlights its potential as a core parental line in breeding new oat varieties with broad environmental adaptability. To assess the varietal performance more accurately, we conducted a comprehensive evaluation using data from both years. The grey rational analysis revealed that PTP had the highest weight coefficient, followed by HD, FC, and L/W. Based on the comprehensive evaluation, the top ten performing varieties were YM49, YM47, YM84, YM48, YM39, YM41, YM79, YM81, YM58, and YM36. For breeding programs aimed at simultaneously improving yield and grain quality, priority should be given to varieties with strong tillering ability, moderate hardness, high fat content, and optimal L/W ratio.

## Conclusions

The 83 naked oat strains we tested exhibited high variability and genetic diversity. Most agronomic and qualitative traits exhibited either a synergistic or conflicting relationships. The strains were classified into three categories. Group I included high-yield, large-grain strains with high GS and GW. These strains could be used as hybrid parents to increase grain quantity and size. Group II included medium-yield, medium-grain strains with prominent TGW. These strains can serve as hybrid parents to enhance grain weight. Group III consisted low-yield, high-nutrition, small-grain strains with prominent L/W, TW, FC, and β-gC. These strains can be used as hybrid parents to improve the nutritional quality of oats. Strains YM49, YM47, YM84, YM48, YM39, YM41, YM79, YM81, YM58, and YM36 are recommended for further production testing. For improvements in yield and quality, cultivators should focus on these synergistic effects rather than on any single trait. The comprehensive effects of other traits should also be considered when selecting materials to achieve specific breeding objectives. Appropriate parents should be selected based on the desired breeding goals with the aim ofcreating optimal hybrid combinations. This approach provides a theoretical foundation for breeding new oats with a high yield and quality.

## Supporting information

S1 Table Differences in mean temperature and rainfall from June to September in 2011 to 2021, 2022 and 2023 (our two test years) Different lowercase letters indicate the difference between different years in the same month
(DOCX)

S2 Table Correlation among different indices within each of the agronomic and qualitative traits of 83 naked oat strains during 2022 to 2023. ** Indicates a significant correlation (P < 0.05), and indicates an extremely significant correlation (P < 0.01).
(DOCX)

S3 Table Comprehensive evaluation of agronomic and qualitative traits of 83 naked oat strains during 2022 to 2023 by use of the membership function. Maecenas convallis mauris sit amet sem ultrices gravida. Etiam eget sapien nibh. Sed ac ipsum eget enim egestas ullamcorper nec euismod ligula. Curabitur fringilla pulvinar lectus consectetur pellentesque.
(DOCX)

S4 Table Correlations of the 2-yr average among agronomic and qualitative traits of 83 naked oat strains. *indicates a significant correlation (P < 0.05), and **indicates an extremely significant correlation (P < 0.01).
(DOCX)
