# Supplementary material for: Screening of agronomic and qualitative physical and chemical traits of 83 naked oats strains
Source: PLoS One. 2025 May 27;20(5):e0324879. doi: 10.1371/journal.pone.0324879 (PMC12111340; doi:10.1371/journal.pone.0324879)
Supplement: S1 Table — Different lowercase letters indicate the difference between different years in the same month (DOCX) [file pone.0324879.s001.docx]

**Supplementary Table 1.** Differences in mean temperature and rainfall from June–September in 2011–2021, and 2022 and 2023 (two test years).

| Climate | Month | 2011–2021 | 2022 | 2023 | Statistics | | |
| --- | --- | --- | --- | --- | --- | --- | --- |
|  |  |  |  |  | *F* | df | *P* |
| Temperature | June | 19.23 ± 0.52 a | 20.57 ± 0.50 a | 19.23 ± 1.24 a | 0.856 | 2, 8 | 0.471 |
|  | July | 21.13 ± 0.13 a | 20.47 ± 0.37 a | 21.43 ± 0.13 a | 4.235 | 2, 8 | 0.071 |
|  | August | 19.23 ± 0.92 a | 19.87 ± 2.17 a | 20.50 ± 2.43 a | 0.082 | 2, 8 | 0.922 |
|  | September | 13.93 ± 1.10 a | 13.80 ± 0.85 a | 15.63 ± 1.88 a | 0.524 | 2, 8 | 0.617 |
| Rainfall | June | 20.70 ± 4.00 a | 19.80 ± 11.83 a | 11.13 ± 5.69 a | 0.655 | 2, 8 | 0.553 |
|  | July | 38.27 ± 5.38 b | 84.20 ± 15.27 a | 25.57 ± 7.89 b | 8.796 | 2, 8 | 0.016 |
|  | August | 38.23 ± 1.92 a | 114.10 ± 47.05 a | 31.77 ± 7.23 a | 3.477 | 2, 8 | 0.099 |
|  | September | 20.33 ± 1.13 a | 19.00 ± 13.17 a | 19.17 ± 6.49 a | 0.124 | 2, 8 | 0.886 |

Different lowercase letters indicate differences between years in the same month.
