# Supplementary material for: Screening of agronomic and qualitative physical and chemical traits of 83 naked oats strains
Source: PLoS One. 2025 May 27;20(5):e0324879. doi: 10.1371/journal.pone.0324879 (PMC12111340; doi:10.1371/journal.pone.0324879)
Supplement: S2 Table — ** Indicates a significant correlation (P < 0.05), and indicates an extremely significant correlation (P < 0.01). (DOCX) [file pone.0324879.s002.docx]

**Supplementary Table 2**. Correlations among different indices within each of the agronomic and qualitative traits of the 83 naked oat strains from 2022 to 2023.

| Agronomy | Year | Thousand grain weight (g) | Productive tillers/plant | Spike length (cm) | Primary spikelets/spike | Grains/spike |  |  |
| --- | --- | --- | --- | --- | --- | --- | --- | --- |
| Productive tillers/plant | 2022 | -0.052 |  |  |  |  |  |  |
|  | 2023 | 0.214 |  |  |  |  |  |  |
|  | 2022-2023 | 0.213 |  |  |  |  |  |  |
| Spike length (cm) | 2022 | -0.128 | -0.092 |  |  |  |  |  |
|  | 2023 | -0.07 | 0.206 |  |  |  |  |  |
|  | 2022-2023 | -0.059 | 0.014 |  |  |  |  |  |
| Primary spikelets/spike | 2022 | -0.359** | 0.141 | 0.254* |  |  |  |  |
|  | 2023 | -0.024 | 0.094 | 0.447** |  |  |  |  |
|  | 2022-2023 | -0.230* | -0.067 | 0.258* |  |  |  |  |
| Grains/spike | 2022 | -0.364** | 0.240* | 0.166 | 0.804** |  |  |  |
|  | 2023 | -0.355** | -0.048 | 0.427** | 0.691** |  |  |  |
|  | 2022-2023 | -0.455** | 0.005 | 0.197 | 0.655** |  |  |  |
| Yield per area (kg) | 2022 | 0.268* | 0.215 | -0.251* | -0.101 | 0.184 |  |  |
|  | 2023 | -0.281* | -0.176 | -0.006 | 0.051 | 0.286** |  |  |
|  | 2022-2023 | -0.096 | 0.036 | -0.17 | -0.153 | 0.218* |  |  |
| Physical quality | Year | Grain length (mm) | Grain width (mm) | Grain area (mm^2^) | Grain perimeter (mm) | Length/width ratio | Test weight (kg) | Specific gravity (g·cm^-3^) |
| Grain width (mm) | 2022 | -0.032 |  |  |  |  |  |  |
|  | 2023 | 0.057 |  |  |  |  |  |  |
|  | 2022-2023 | -0.103 |  |  |  |  |  |  |
| Grain area (mm^2^) | 2022 | 0.628** | 0.734** |  |  |  |  |  |
|  | 2023 | 0.781** | 0.651** |  |  |  |  |  |
|  | 2022-2023 | 0.698** | 0.623** |  |  |  |  |  |
| Grain perimeter (mm) | 2022 | 0.955** | 0.091 | 0.650** |  |  |  |  |
|  | 2023 | 0.988** | 0.126 | 0.811** |  |  |  |  |
|  | 2022-2023 | 0.979** | 0.014 | 0.751** |  |  |  |  |
| Length/width ratio | 2022 | 0.717** | -0.716** | -0.076 | 0.603** |  |  |  |
|  | 2023 | 0.775** | -0.584** | 0.221* | 0.726** |  |  |  |
|  | 2022-2023 | 0.791** | -0.688** | 0.124 | 0.705** |  |  |  |
| Test weight (kg) | 2022 | -0.461** | 0.503** | 0.111 | -0.381** | -0.680** |  |  |
|  | 2023 | -0.730** | 0.201 | -0.427** | -0.715** | -0.715** |  |  |
|  | 2022-2023 | -0.623** | 0.402** | -0.18 | -0.578** | -0.704** |  |  |
| Specific gravity (g·cm^-3^) | 2022 | -0.013 | -0.081 | -0.087 | 0.026 | 0.041 | 0.443** |  |
|  | 2023 | -0.155 | -0.117 | -0.188 | -0.17 | -0.05 | 0.390** |  |
|  | 2022-2023 | -0.042 | -0.145 | -0.156 | -0.048 | 0.058 | 0.399** |  |
| Degree of hardness | 2022 | -0.601** | 0.111 | -0.315** | -0.585** | -0.486** | 0.092 | -0.290** |
|  | 2023 | -0.620** | 0.319** | -0.278* | -0.609** | -0.696** | 0.481** | 0.024 |
|  | 2022-2023 | -0.749** | 0.18 | -0.451** | -0.730** | -0.648** | 0.343** | -0.141 |
| Chemical quality | Year | Fat content (%) | Protein content (%) | Total starch content (%) | Water content (%) |  |  |  |
| Protein content (%) | 2022 | -0.189 |  |  |  |  |  |  |
|  | 2023 | -0.049 |  |  |  |  |  |  |
|  | 2022-2023 | -0.068 |  |  |  |  |  |  |
| Total starch content (%) | 2022 | 0.045 | -0.723** |  |  |  |  |  |
|  | 2023 | -0.038 | -0.891** |  |  |  |  |  |
|  | 2022-2023 | -0.031 | -0.796** |  |  |  |  |  |
| Water content (%) | 2022 | -0.627** | 0.332** | -0.294** |  |  |  |  |
|  | 2023 | -0.720** | 0.208 | -0.358** |  |  |  |  |
|  | 2022-2023 | -0.715** | 0.240* | -0.273* |  |  |  |  |
| β-glucan content (%) | 2022 | 0.261* | -0.232* | 0.095 | -0.203 |  |  |  |
|  | 2023 | 0.344** | -0.165 | 0.12 | -0.358** |  |  |  |
|  | 2022-2023 | 0.547** | -0.303** | 0.14 | -0.478** |  |  |  |

* Indicates a significant correlation (*P* < 0.05), and ** indicates a highly significant correlation (*P* < 0.01)
