# Supplementary material for: Screening of agronomic and qualitative physical and chemical traits of 83 naked oats strains
Source: PLoS One. 2025 May 27;20(5):e0324879. doi: 10.1371/journal.pone.0324879 (PMC12111340; doi:10.1371/journal.pone.0324879)
Supplement: S3 Table — Maecenas convallis mauris sit amet sem ultrices gravida. Etiam eget sapien nibh. Sed ac ipsum eget enim egestas ullamcorper nec euismod ligula. Curabitur fringilla pulvinar lectus consectetur pellentesque. (DOCX) [file pone.0324879.s003.docx]

**Supplementary Table 3.** Comprehensive evaluation of the agronomic and qualitative traits of 83 naked oat strains from 2022 to 2023 using the membership function.

| Materials | Agronomy | | Physical quality | | Chemical Quality | | Agronomy & Quality | |
| --- | --- | --- | --- | --- | --- | --- | --- | --- |
|  | Score | rank | Score | Rank | Score | Rank | Score | Rank |
| YM2 | 0.559 | 9 | 0.765 | 8 | 0.328 | 55 | 0.675 | 7 |
| YM3 | 0.268 | 77 | 0.592 | 52 | 0.321 | 56 | 0.527 | 57 |
| YM5 | 0.567 | 8 | 0.665 | 34 | 0.268 | 61 | 0.588 | 36 |
| YM6 | 0.528 | 16 | 0.681 | 26 | 0.201 | 69 | 0.586 | 38 |
| YM7 | 0.555 | 11 | 0.624 | 42 | 0.279 | 60 | 0.579 | 39 |
| YM8 | 0.300 | 68 | 0.727 | 16 | 0.158 | 76 | 0.570 | 43 |
| YM9 | 0.179 | 83 | 0.608 | 47 | 0.124 | 80 | 0.495 | 64 |
| YM11 | 0.559 | 10 | 0.321 | 78 | 0.220 | 67 | 0.322 | 79 |
| YM12 | 0.453 | 34 | 0.370 | 74 | 0.225 | 66 | 0.306 | 81 |
| YM13 | 0.247 | 81 | 0.750 | 13 | 0.557 | 11 | 0.587 | 37 |
| YM14 | 0.526 | 18 | 0.558 | 60 | 0.371 | 48 | 0.578 | 40 |
| YM15 | 0.405 | 40 | 0.590 | 54 | 0.342 | 54 | 0.528 | 56 |
| YM16 | 0.574 | 7 | 0.615 | 45 | 0.574 | 10 | 0.644 | 14 |
| YM17 | 0.471 | 31 | 0.700 | 22 | 0.577 | 9 | 0.644 | 13 |
| YM18 | 0.520 | 19 | 0.667 | 33 | 0.532 | 20 | 0.624 | 18 |
| YM19 | 0.344 | 53 | 0.664 | 35 | 0.619 | 6 | 0.610 | 29 |
| YM20 | 0.379 | 45 | 0.678 | 27 | 0.628 | 3 | 0.620 | 20 |
| YM21 | 0.369 | 49 | 0.758 | 9 | 0.461 | 33 | 0.625 | 17 |
| YM22 | 0.342 | 54 | 0.790 | 4 | 0.187 | 73 | 0.630 | 15 |
| YM23 | 0.301 | 67 | 0.861 | 1 | 0.191 | 71 | 0.668 | 8 |
| YM24 | 0.318 | 63 | 0.663 | 36 | 0.216 | 68 | 0.578 | 41 |
| YM25 | 0.474 | 30 | 0.803 | 3 | 0.106 | 82 | 0.653 | 12 |
| YM26 | 0.292 | 72 | 0.777 | 7 | 0.188 | 72 | 0.612 | 27 |
| YM27 | 0.369 | 48 | 0.756 | 10 | 0.178 | 74 | 0.598 | 34 |
| YM28 | 0.362 | 50 | 0.788 | 5 | 0.251 | 62 | 0.626 | 16 |
| YM29 | 0.485 | 26 | 0.667 | 32 | 0.115 | 81 | 0.510 | 59 |
| YM30 | 0.335 | 59 | 0.716 | 19 | 0.162 | 75 | 0.541 | 49 |
| YM31 | 0.457 | 33 | 0.755 | 11 | 0.080 | 83 | 0.602 | 32 |
| YM32 | 0.443 | 35 | 0.638 | 38 | 0.345 | 53 | 0.535 | 51 |
| YM33 | 0.717 | 2 | 0.736 | 14 | 0.291 | 58 | 0.654 | 11 |
| YM34 | 0.370 | 47 | 0.341 | 76 | 0.346 | 52 | 0.373 | 75 |
| YM35 | 0.381 | 43 | 0.269 | 80 | 0.475 | 30 | 0.349 | 77 |
| YM36 | 0.499 | 22 | 0.201 | 82 | 0.468 | 32 | 0.304 | 82 |
| YM37 | 0.355 | 52 | 0.290 | 79 | 0.449 | 36 | 0.315 | 80 |
| YM38 | 0.494 | 23 | 0.807 | 2 | 0.143 | 78 | 0.658 | 9 |
| YM39 | 0.489 | 25 | 0.672 | 28 | 0.813 | 1 | 0.690 | 3 |
| YM41 | 0.478 | 28 | 0.783 | 6 | 0.623 | 5 | 0.791 | 1 |
| YM42 | 0.303 | 66 | 0.725 | 17 | 0.578 | 8 | 0.680 | 4 |
| YM43 | 0.380 | 44 | 0.731 | 15 | 0.598 | 7 | 0.700 | 2 |
| YM44 | 0.360 | 51 | 0.671 | 29 | 0.416 | 44 | 0.618 | 22 |
| YM45 | 0.310 | 64 | 0.622 | 43 | 0.431 | 39 | 0.575 | 42 |
| YM46 | 0.221 | 82 | 0.668 | 31 | 0.557 | 12 | 0.612 | 26 |
| YM47 | 0.462 | 32 | 0.592 | 51 | 0.473 | 31 | 0.604 | 31 |
| YM48 | 0.491 | 24 | 0.601 | 48 | 0.453 | 35 | 0.624 | 19 |
| YM49 | 0.728 | 1 | 0.555 | 63 | 0.546 | 18 | 0.679 | 6 |
| YM50 | 0.427 | 38 | 0.577 | 55 | 0.553 | 15 | 0.615 | 24 |
| YM51 | 0.326 | 61 | 0.548 | 65 | 0.490 | 28 | 0.530 | 54 |
| YM52 | 0.554 | 12 | 0.625 | 41 | 0.552 | 16 | 0.607 | 30 |
| YM53 | 0.265 | 79 | 0.755 | 12 | 0.409 | 45 | 0.618 | 21 |
| YM55 | 0.500 | 20 | 0.538 | 66 | 0.538 | 19 | 0.595 | 35 |
| YM56 | 0.275 | 75 | 0.576 | 56 | 0.455 | 34 | 0.479 | 67 |
| YM57 | 0.579 | 6 | 0.331 | 77 | 0.556 | 14 | 0.356 | 76 |
| YM58 | 0.537 | 15 | 0.494 | 70 | 0.424 | 40 | 0.504 | 61 |
| YM59 | 0.342 | 55 | 0.465 | 71 | 0.439 | 38 | 0.422 | 72 |
| YM61 | 0.545 | 13 | 0.496 | 69 | 0.549 | 17 | 0.542 | 48 |
| YM62 | 0.479 | 27 | 0.536 | 67 | 0.509 | 23 | 0.548 | 47 |
| YM63 | 0.437 | 37 | 0.592 | 53 | 0.509 | 24 | 0.555 | 46 |
| YM65 | 0.289 | 73 | 0.712 | 20 | 0.447 | 37 | 0.525 | 58 |
| YM67 | 0.340 | 56 | 0.699 | 23 | 0.370 | 49 | 0.610 | 28 |
| YM68 | 0.270 | 76 | 0.608 | 46 | 0.396 | 46 | 0.455 | 69 |
| BY-13 | 0.300 | 69 | 0.433 | 72 | 0.247 | 64 | 0.415 | 73 |
| YM71 | 0.278 | 74 | 0.619 | 44 | 0.128 | 79 | 0.530 | 55 |
| YM72 | 0.327 | 60 | 0.721 | 18 | 0.626 | 4 | 0.680 | 5 |
| YM73 | 0.476 | 29 | 0.359 | 75 | 0.691 | 2 | 0.441 | 71 |
| YM74 | 0.544 | 14 | 0.557 | 61 | 0.422 | 41 | 0.534 | 53 |
| YM75 | 0.392 | 42 | 0.645 | 37 | 0.353 | 51 | 0.599 | 33 |
| JY-8 | 0.293 | 71 | 0.555 | 64 | 0.491 | 27 | 0.500 | 62 |
| YM76 | 0.339 | 58 | 0.564 | 58 | 0.286 | 59 | 0.454 | 70 |
| YM77 | 0.499 | 21 | 0.683 | 24 | 0.248 | 63 | 0.537 | 50 |
| YM78 | 0.323 | 62 | 0.599 | 49 | 0.239 | 65 | 0.473 | 68 |
| YM79 | 0.443 | 36 | 0.670 | 30 | 0.512 | 22 | 0.613 | 25 |
| YM80 | 0.339 | 57 | 0.597 | 50 | 0.480 | 29 | 0.563 | 45 |
| YM81 | 0.416 | 39 | 0.682 | 25 | 0.420 | 42 | 0.568 | 44 |
| BY-3 | 0.526 | 17 | 0.238 | 81 | 0.492 | 25 | 0.348 | 78 |
| YM82 | 0.392 | 41 | 0.567 | 57 | 0.143 | 77 | 0.491 | 65 |
| YM83 | 0.677 | 4 | 0.380 | 73 | 0.318 | 57 | 0.481 | 66 |
| YM84 | 0.685 | 3 | 0.711 | 21 | 0.491 | 26 | 0.657 | 10 |
| YM85 | 0.585 | 5 | 0.631 | 39 | 0.557 | 13 | 0.616 | 23 |
| YM87 | 0.248 | 80 | 0.629 | 40 | 0.198 | 70 | 0.535 | 52 |
| YM91 | 0.267 | 78 | 0.562 | 59 | 0.365 | 50 | 0.505 | 60 |
| YM92 | 0.307 | 65 | 0.535 | 68 | 0.418 | 43 | 0.380 | 74 |
| YM94 | 0.297 | 70 | 0.162 | 83 | 0.530 | 21 | 0.293 | 83 |
| YM96 | 0.379 | 46 | 0.556 | 62 | 0.375 | 47 | 0.495 | 63 |
