# Supplementary material for: Screening of agronomic and qualitative physical and chemical traits of 83 naked oats strains
Source: PLoS One. 2025 May 27;20(5):e0324879. doi: 10.1371/journal.pone.0324879 (PMC12111340; doi:10.1371/journal.pone.0324879)
Supplement: S4 Table — *indicates a significant correlation (P < 0.05), and **indicates an extremely significant correlation (P < 0.01). (DOCX) [file pone.0324879.s004.docx]

**Supplementary Table 4**. Correlations of the two-year average among agronomic and qualitative traits of 83 naked oat strains.

| Traits | | Agronomy | | | | | | Physical quality | | | | | | | | Chemical Quality | | | |
| --- | --- | --- | --- | --- | --- | --- | --- | --- | --- | --- | --- | --- | --- | --- | --- | --- | --- | --- | --- |
|  |  | Thousand grain weight (g) | Productive tillers/plant | Spike length (cm) | Primary spikelets/spike | Grains/spike | Yield per area (kg) | Grain length (mm) | Grain width (mm) | Grain area (mm^2^) | Grain perimeter (mm) | Length/width ratio | Test weight (kg) | Specific gravity (g·cm^-3^) | Degree of hardness | Fat content (%) | Protein content (%) | Total starch content (%) | Water content (%) |
| Agronomy | Thousand grain weight (g) | 0.213 |  |  |  |  |  |  |  |  |  |  |  |  |  |  |  |  |  |
|  | Spike length (cm) | -0.059 | 0.014 |  |  |  |  |  |  |  |  |  |  |  |  |  |  |  |  |
|  | Primary spikelets/spike | -0.230* | -0.067 | 0.258* |  |  |  |  |  |  |  |  |  |  |  |  |  |  |  |
|  | Grains/spike | -0.455** | 0.005 | 0.197 | 0.655** |  |  |  |  |  |  |  |  |  |  |  |  |  |  |
|  | Yield per area (kg) | -0.096 | 0.036 | -0.172 | -0.153 | 0.218* |  |  |  |  |  |  |  |  |  |  |  |  |  |
| Physical quality | Grain length (mm) | 0.401** | 0.005 | -0.317** | 0.048 | 0.165 | 0.165 |  |  |  |  |  |  |  |  |  |  |  |  |
|  | Grain width (mm) | 0.732** | 0.378** | 0.115 | -0.245* | -0.476** | -0.125 | -0.103 |  |  |  |  |  |  |  |  |  |  |  |
|  | Grain area (mm^2^) | 0.809** | 0.306** | -0.126 | -0.153 | -0.2 | 0.065 | 0.698** | 0.623** |  |  |  |  |  |  |  |  |  |  |
|  | Grain perimeter (mm) | 0.522** | 0.048 | -0.301** | -0.013 | 0.091 | 0.165 | 0.979** | 0.014 | 0.751** |  |  |  |  |  |  |  |  |  |
|  | Length/width ratio | -0.151 | -0.227* | -0.301** | 0.192 | 0.409** | 0.181 | 0.791** | -0.688** | 0.124 | 0.705** |  |  |  |  |  |  |  |  |
|  | Test weight (kg) | 0.131 | 0.214 | 0.254* | -0.215 | -0.367** | -0.035 | -0.623** | 0.402** | -0.18 | -0.578** | -0.704** |  |  |  |  |  |  |  |
|  | Specific gravity (g·cm^-3^) | 0 | -0.16 | 0.028 | 0.089 | -0.05 | -0.06 | -0.042 | -0.145 | -0.156 | -0.048 | 0.058 | 0.399** |  |  |  |  |  |  |
|  | Degree of hardness | -0.219* | -0.115 | 0.333** | 0.125 | -0.087 | -0.313** | -0.749** | 0.18 | -0.451** | -0.730** | -0.648** | 0.343** | -0.141 |  |  |  |  |  |
| Chemical quality | Fat content (%) | -0.051 | 0.199 | 0.222* | -0.074 | 0.109 | 0.096 | -0.260* | 0.09 | -0.124 | -0.238* | -0.248* | 0.178 | -0.2 | 0.233* |  |  |  |  |
|  | Protein content (%) | -0.008 | -0.14 | 0.135 | 0.098 | -0.173 | -0.471** | -0.153 | -0.071 | -0.215 | -0.148 | -0.055 | 0.076 | 0.204 | 0.183 | -0.068 |  |  |  |
|  | Total starch content (%) | 0.031 | 0.156 | -0.079 | -0.034 | 0.115 | 0.218* | 0.063 | 0.167 | 0.212 | 0.04 | -0.063 | -0.002 | -0.227* | -0.069 | -0.031 | -0.796** |  |  |
|  | Water content (%) | -0.175 | -0.151 | -0.155 | 0.14 | 0.041 | 0.03 | 0.124 | -0.330** | -0.154 | 0.098 | 0.291** | -0.153 | 0.225* | -0.145 | -0.715** | 0.240* | -0.273* |  |
|  | β-glucan content (%) | -0.136 | 0.129 | 0.278* | 0.014 | 0.159 | 0.031 | -0.213 | -0.002 | -0.149 | -0.2 | -0.153 | 0.006 | -0.101 | 0.259* | 0.547** | -0.303** | 0.14 | -0.478** |

* indicates a significant correlation (*P* < 0.05), and ** indicates a highly significant correlation (*P* < 0.01)
